# Application of geographical information system in disposal site selection for hazardous wastes

**DOI:** 10.1186/s40201-014-0141-3

**Published:** 2014-11-28

**Authors:** Mehdi Rezaeimahmoudi, Abdolreza Esmaeli, Alireza Gharegozlu, Hassan Shabanian, Ladan Rokni

**Affiliations:** Department of GIS & RS, School of Environment and Energy , Science and Research Branch, Islamic Azad University, Tehran, Iran; Plasma and Nuclear Fusion Research School, Nuclear Science and Technology Research Institute, AEOI, Tehran, Iran; Department of GIS & RS, School of Geography, University of Tehran, Tehran, Iran; Department of Tourism Planning, School of Human Geography, University of Tehran, Tehran, Iran

**Keywords:** Geographical information system (GIS), Disposal site selection, Hazardous waste, AHP, Locating criteria, Iran

## Abstract

**Background:**

The aim of this study was to provide a scientific method based on Geographical Information System (GIS) regarding all sustainable development measures to locate a proper landfill for disposal of hazardous wastes, especially industrial (radioactive) wastes.

**Methods:**

Seven effective factors for determining hazardous waste landfill were applied in Qom Province, central Iran. These criteria included water, slope, population centers, roads, fault, protected areas and geology. The Analysis Hierarchical Process (AHP) model based on pair comparison was used. First, the weight of each factor was determined by experts; afterwards each layer of maps entered to ARC GIS and with special weight multiplied together, finally the best suitable site was introduced.

**Results:**

The most suitable sites for burial were in northwest and west of Qom Province and eventually five zones were introduced as the sample sites.

**Conclusion:**

GIs and AHP model is introduced as the technical, useful and accelerator tool for disposal site selection. Furthermore it is determined that geological factor is the most effective layer for site selection. It is suggested that geological conditions should be considered primarily then other factors are taken into consideration.

## Introduction

Protecting human health and the environment in a world rapidly becoming more and more complicated and considered as a major challenge for the international community. Developing in technology and industry leaves some materials, which are hazardous for human life. These materials are potentially dangerous or later cause some risks in environment.

There are some materials used by refineries, power factories, chemical and petrochemical industries that create hazardous wastes. In general, experts recognize that hazardous materials and waste are resulted from the activities of various sectors such as industry, agriculture, trade, and services [1]. Rosenfeld believes that” If a material has been discarded and can cause substantial harm to humans or the environment, it is considered a hazardous waste” [2]. Additionally, hazardous material is, by another definition, “a matter which is itself hazardous for human, animal, and environment health” [3]. According to the suggested definition by Iran Department of Environment (IDOE), hazardous wastes are wastes, which have one of the properties, including toxic, flammable, corrosive and pathogenic – for human, animal, or environment [4].

Radioactive materials are used in medicine, research centers and operation of nuclear power plants, and fuel recycling lead to the production of radioactive wastes, which has negative effect on the surrounding environment. Now, more than 50 countries have consumed their stored fuel and are waiting for reprocessing or disposal them [5]. Various approaches have been investigated in different countries, such as application of iron nanaoparticles in landfill leachate treatment [6], or Converting them into a value added product [7] for usual waste; and for dangerous waste: burying radioactive wastes in shallow pits, waste disposal deep underground, burial in halite formations, burial and disposal at sea and in space. Remaining these wastes has been a challenge and global growing issue, hence finding a suitable site for burial them is being deterioration because there are numerous factors and parameters involved. Besides, it is a complex, costing and time-consuming issue.

It is necessary for burial sites to have some geological characteristics. Based on the IDOE Standards these sites should contain the following items: [8-10]

- Suitable substrate of low permeability and high strength with minimal groundwater trend
- Homogeneous rock mass slope with low and stable hydraulic steep,
- Having a low vibration with low frequency
- Stay away from earthquake fault line
- Stay away from populated areas and protected areas and national reserves
- Stay away from lakes and rivers and groundwater discharge areas

Although no research has been done on nuclear technology due to its novelty in Iran, but there are some studies on hazardous wastes, which the common point among all of them is using GIS techniques to locating a proper site for wastes disposal [11-13]. Similarly, GIS techniques and Analysis Hierarchical Process (AHP) has combined to select the best disposal location [14] and an environmental approach was applied to provide the disposal measures [15]. In other countries, some researches in this field have been conducted using GIS; for instance it is said that GIS has a good accuracy to reduce the errors in spatial databases [16]. Furthermore, GIS have been used for the suitability analysis of nuclear waste [17].

The aim of this study was to provide a scientific method, and prevent of risks, occurred due to incorrect disposal site selection. This selection should be done based on the specific circumstance of each area. To clarify the issue, new devices have been established, among which, geographical information system (GIS) is of great prominence. This tool can introduce a suitable site for burning waste with spending less time and money using spatial data. This study focused on industrial (radioactive) wastes.

## Materials and methods

### Study area

**Figure 1:**
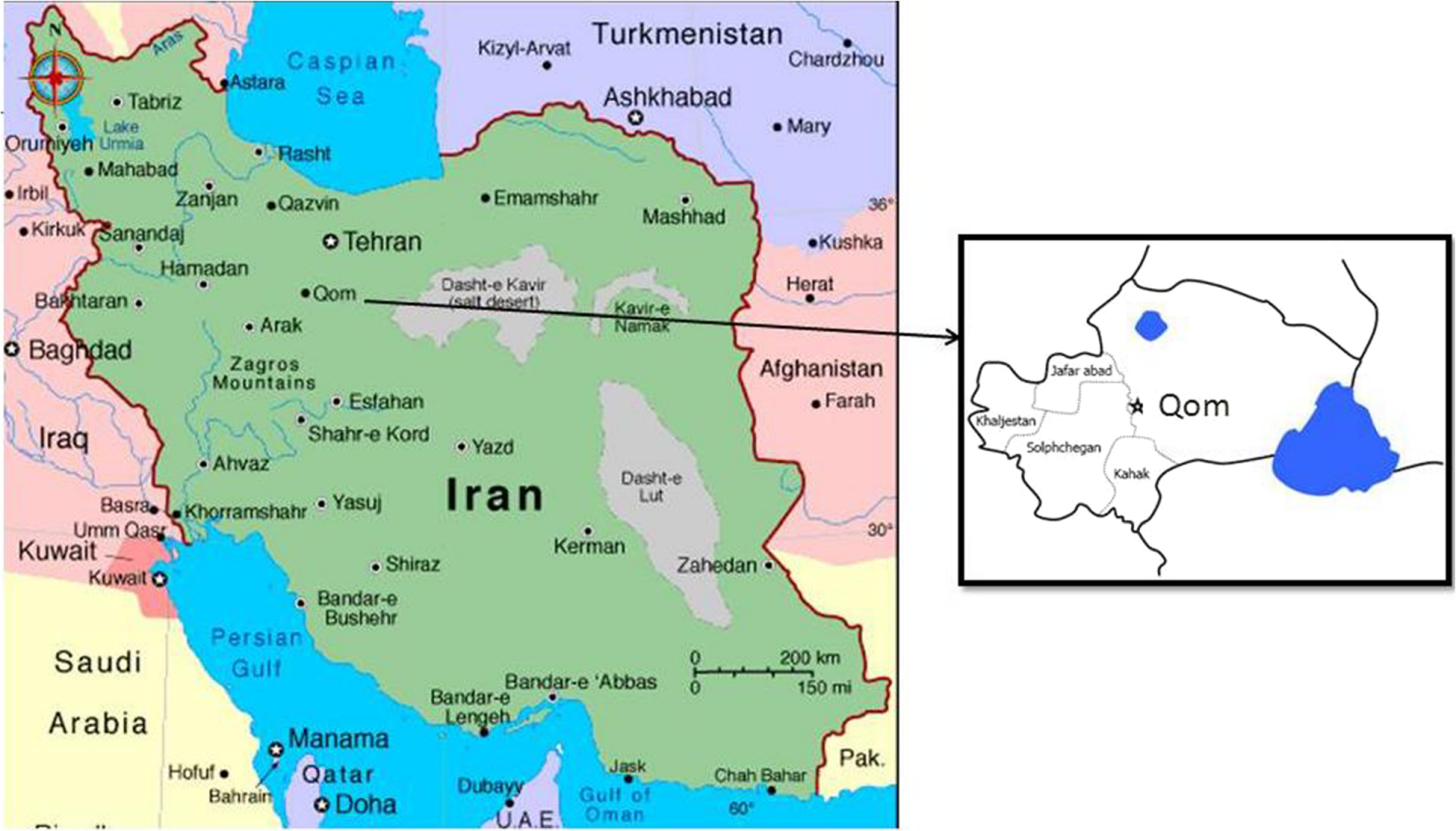
**Location of Qom Province, central Iran.**

The Qom Province, central Iran is located at the west of Salt Lack (Figure 1). Area of this province is 0.68% of Iran [18].

### Data collection

A compendium of methods were implemented to proceed the study as follows: determining the measures for identifying disposal location, reviewing relevant previous studies, study of review articles by the relevant international organizations, and interviews with experts in environmental organizations and Nuclear Energy Organization of Iran.

With reconciliation of the obtained data, the most effective measures based on the studying area were determined, including water resources, slope, population centers, communication lines, protected zones, fault and geology. The measures for mapping were obtained from data of various agencies, such as topographic maps scaled 1/50000 of National Cartographic Center (NCC), geological maps scaled 1/250000, the map of political divisions and rural and urban areas of the country, the map of protected areas of IDOE, provincial road maps provided by Ministry of Road and Urban Development, and ETM images of LandSat. Much of the collected information was raw data, which converted into useful date by using information processing methods of various software packages, including ARCGIS and ENVI. Since the AHP model [19] has been used in the study. Delphi questionnaire was prepared to determine the weights of measures, which was completed by experts related to each measure and incorporated into the final weights.

### Data analysis

Base maps were prepared by using GIS & RS analysts, then replaced with weights determined by the experts using AHP model, and finally the appropriate sites were determined based on the identified priorities.

### Positioning

The environment is a complex system, which needs a model to investigate all its aspect, and influencing factors and sub-factors [20]. To advance this goal, by reviewing the organizational resources, information, and procedures, and despite all limitations, some measures have been identified.

**Figure 2:**
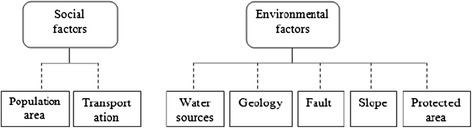
**Studied criterias and sub criterias.**

The measures were divided into environmental measures and social measures, which have their own sub-measures; moreover, the sub-measures were also interrelated while there were associations between two classes (Figure 2).

### Ethical approval

The study was approved by the Ethics Committee of Science and Research Branch, Islamic Azad University, Tehran, number 92290.

## Results

To choose the landfill, we adopted a holistic, in which quantitative methods were used for concluding. Selected measures were divided into two major areas based on research problem: Environmental and socioeconomic as shown in Table 1. Since the environmental measures for locating the landfill are more important, they were assigned higher weights and more important than others. The weights were set according to the experts’ idea. Table 1 indicates the method of determining measure in the study using comparative deductions of previous studies [16,17,21,22].

**Table 1:** **Determining the criteria for disposal site selection of hazardous wastes using previous studies** [16,17,21,22]

**Reference No.**				
**[** 16 **]**	**[** 17 **]**	**[** 21 **]**	**[** 22 **]**	**Present study**
Geology	Slope	Heidrology	Geology	Geology
Transportation	Geology	Geology	Accessibility	Slope
Environment	Protected areas	Environmental	Population Distribution	Protected areas
Population	Water source	Technical	Hydrology	Water source
	Population centers	Climate		Population centers
	Communication lines	Social		Communication lines
				Faults

After comparing pair-wise environmental and socioeconomic measures based on AHP method [23], each expert evaluated independently measures of research problem, and calculated the final weight by using approximate arithmetic mean methods (Table 2).

**Table 2:** **The expert’s opinion about the criteria’s weight based on AHP method**

	**Expert 1**	**Expert 2**	**Expert 3**	**Expert 4**	**Weight**
**Geology**	0.287305	0.242083	0.260185	0.26766	0.264308
**Slope**	0.152447	0.159808	0.178881	0.153448	0.161146
**Population centers**	0.103835	0.117281	0.108006	0.118455	0.111894
**Protected areas**	0.107581	0.100433	0.096106	0.09548	0.0999
**Water source**	0.118128	0.115308	0.106863	0.128787	0.117272
**Distance from road**	0.106083	0.097759	0.09382	0.09359	0.097813
**Faults**	0.124621	0.167328	0.156139	0.14258	0.147667
**Total**	1	1	1	1	1

After determining weights and preparing standard maps of selected measures, all layers incorporated into the final map were created in the ArcGIS software based on the following formula.

$$ R=\left({\displaystyle \sum_{c=1}^{nc}{W}_C{C}_C}\right) $$

Were, C is: Criteria, NC: Number of Criteria, WC: The weight of each criterion, C_C:_ Scores for each criterion.

**Figure 3:**
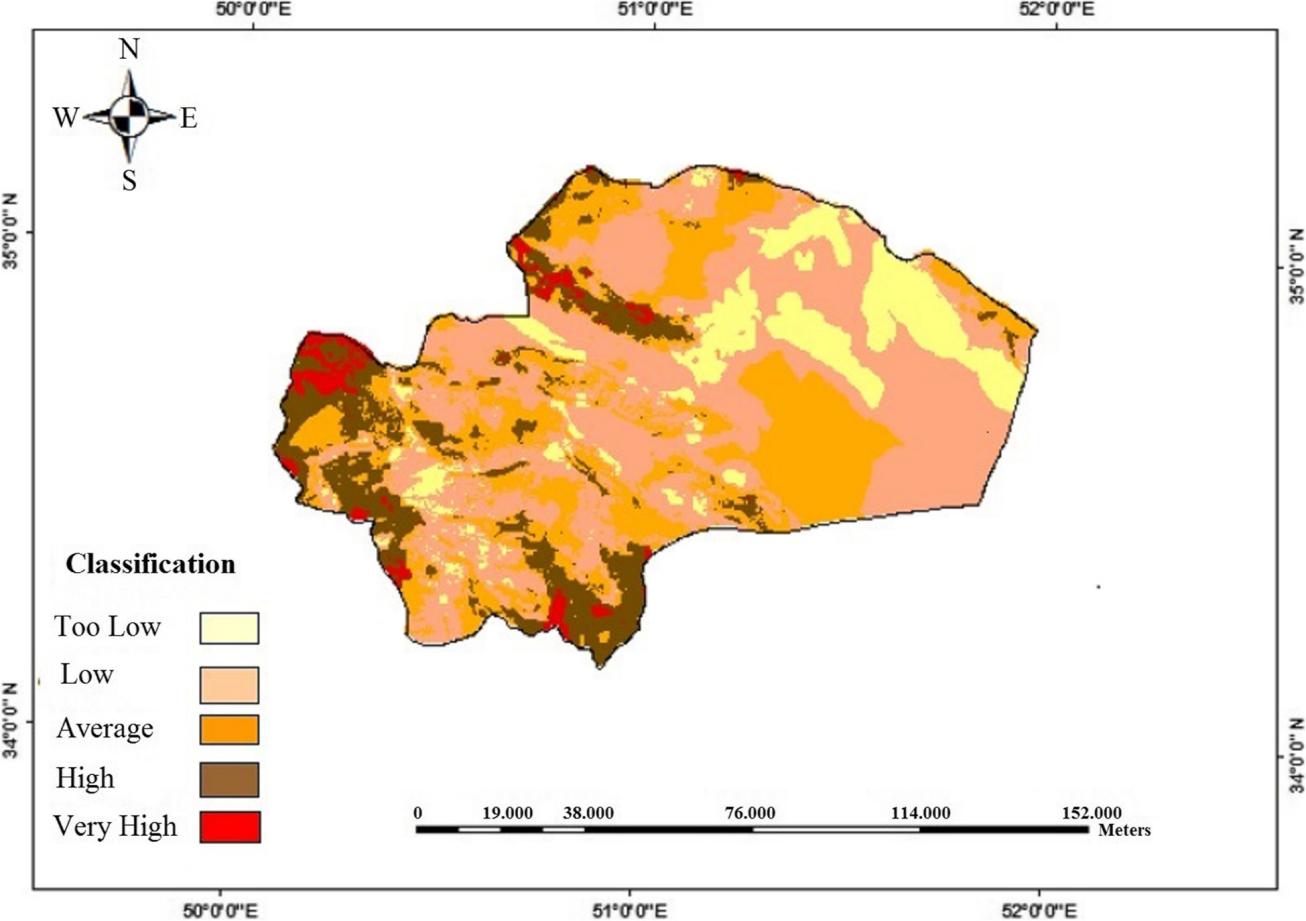
**Zoning of appropriate locations for disposal wastes in the region.**

Results indicated that the studied province had relatively proper situation for disposal of nuclear wastes in terms of six criteria of water resources, slope, population centers, roads, protected areas and fault. However, given the constraints of the studied area in relation to geological formations, the map indicated that very limited areas in the north, northwest, southwest and south of Qom Province are appropriate for disposal of nuclear wastes; means that geological measure is considered as the most important limiting factor in the identification of nuclear waste disposal in the city of Qom. Figure 3 shows the most appropriate locations for disposal wastes in the region.

## Discussion

In this study, we could introduce a technical and useful tool for Disposal Site Selection.

It is believed that site selection is the vital part of burying hazardous waste. This stage, known as the final stage of hazardous waste management, is very complex and technologically demanding [2]. Chen et al. considers the impact of environmental, social and economic problems, caused by burying disposals, as the considerable parts [24].

Our results showed that GIS could be used as an analyst in site selecting for nuclear waste disposal. Using GIS, similar studies have been conducted in other countries, for example NIREX (Nuclear Industry Radioactive Waste Executive, UK), which is a British company with responsibility for identifying radioactive waste disposal site, did a useful investigation and concluded that GIS was an accelerator tool for reaching the goal in this arena [25].

Furthermore, considering seven main criteria in this paper, and using them in incorporation of AHP model and GIS, lead us to understand that selected area in Qom Province has limited potential based on geology; therefore, very limited areas of Qom surface are suitable for nuclear waste disposal. In a relevant study done by Openshaw, using GIS, at first, some main layers were determined, then he tried to choose the most effective layer for site selection, which finally found the geology layer as the most important one [16]. Furthermore, it is confirmed that all criteria related to geology have the most effective role [26]. On the other hand AHP model is introduced as an auxiliary and functional tool in combination, rating and disposal site selection [17]. Combine GIS, AHP in Serbia as a strategic approach to waste management has replaced with traditional methods [27].

## Conclusion

Geological factor is introduced as the most important element in disposal site selection for hazardous waste and since it acts like a limiting factor, we suggest that firstly geological conditions should be examined and in next steps other factors be considered. In addition, Landfill sites should be located in homogeneous bedrock, Lands that are less permeable and where groundwater levels are low.

The main limitation in such investigation is obtaining necessary base information. Consequently need for comprehensive research is undeniable in this arena.
